# Pushing CT and MR Imaging to the Molecular Level for Studying the “Omics”: Current Challenges and Advancements

**DOI:** 10.1155/2014/365812

**Published:** 2014-03-13

**Authors:** Hsuan-Ming Huang, Yi-Yu Shih

**Affiliations:** Healthcare Sector, Siemens Limited Taiwan, 2F No. 3, Yuan Qu Street, Nan Gang District, Taipei 11503, Taiwan

## Abstract

During the past decade, medical imaging has made the transition from anatomical imaging to functional and even molecular imaging. Such transition provides a great opportunity to begin the integration of imaging data and various levels of biological data. In particular, the integration of imaging data and multiomics data such as genomics, metabolomics, proteomics, and pharmacogenomics may open new avenues for predictive, preventive, and personalized medicine. However, to promote imaging-omics integration, the practical challenge of imaging techniques should be addressed. In this paper, we describe key challenges in two imaging techniques: computed tomography (CT) and magnetic resonance imaging (MRI) and then review existing technological advancements. Despite the fact that CT and MRI have different principles of image formation, both imaging techniques can provide high-resolution anatomical images while playing a more and more important role in providing molecular information. Such imaging techniques that enable single modality to image both the detailed anatomy and function of tissues and organs of the body will be beneficial in the imaging-omics field.

## 1. Transition from Morphological to Molecular Imaging

Over the past few decades, imaging data have been widely used to study how body system work together at the organ-tissue level. In contrast, omics data obtained from genomics, metabolomics, proteomics, and pharmacogenomics have been used to provide biological information at the cellular and molecular level. Such omics data may provide a great opportunity to understand the development and progression of diseases. With the integration of imaging and omics data, scientists may find new insights into the mechanism of disease and its treatment. To integrate images as part of the omics, imaging techniques are needed to be safe, fast, accurate and convenient. More importantly, these techniques should have the ability to provide anatomical and functional information.

Among several imaging techniques, computed tomography (CT) and magnetic resonance imaging (MRI) have the potential to satisfy the above requirements. First, human anatomical structures can be imaged using both CT and MRI. Second, recent studies showed that genomics data were correlated with functional (molecular) CT and MRI data [1–8]. It is noteworthy that MR spectroscopy is a conventional technology which could approach in vivo molecular and metabolism information, and there are many valuable results combined with various omics data in different clinical applications, such as oncology and neural diseases, in recent decades [9–12]. Moreover, several nanoparticles such as gold nanoparticles [13, 14] and polymer-coated bismuth sulfide nanoparticles [15] have emerged as novel CT contrast agents for cancer detection. Similarly, some novel genetically encoded MRI contrast agents and advanced sequence technologies have been used to detect gene expression [16–19]. Finally, due to the advantages of cost, convenience (for CT), and safety (for MRI), both CT and MRI will be appropriate imaging techniques for imaging-omics integration.

To build and validate the relationship between omics data and imaging features, there are several limitations that need to be considered. In CT, both anatomical and functional data can be obtained within a few minutes. However, multiple CT scans are required, and patients would receive a high dose of radiation that may increase the risk of developing cancer. As a result, dose reduction in CT is an important issue for the integration between CT and omics data. Unlike CT, MRI does not use ionizing radiation. However, it takes a relatively long acquisition time to obtain anatomical and functional data and may not be acceptable for uncooperative subjects. Thus, acceleration is an important issue for the integration between MRI and omics data. Since the radiation dose of CT and acquisition time of MRI can determine the quantity and quality of anatomical and functional data, they are the key to imaging-omics integration. Hence, the aim of this paper is to review existing technological strategies and new directions that are used for reducing radiation dose in CT and acquisition time in MRI.

## 2. Dose Reduction in Computer Tomography

**Figure 1:**
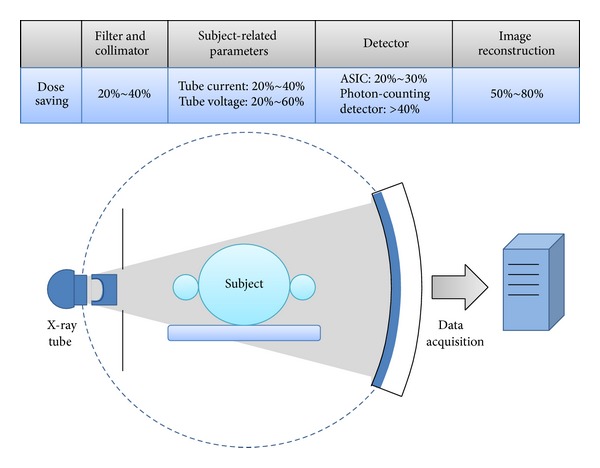
The key component of a CT system and the dose-saving potential of each component.

Nowadays, CT is one of the most commonly used imaging modalities because CT scanning is simple, fast, noninvasive, and accurate. Despite the tremendous benefits of CT scans, radiation dose from CT scans may be a potential risk factor for cancer development. In order to keep radiation as low as reasonably achievable (ALARA), techniques and strategies for reducing patient dose are highly desirable. We would discuss the dose-saving strategies of each component in a CT system shown in Figure 1.

### 2.1. CT Data Acquisition and Processing

#### 2.1.1. Beam Filtration

Generally, X-ray beam emitted from an X-ray tube contains a spectrum of photon energies. Among these X-rays, soft (i.e., low-energy) X-rays are not hard enough to efficiently penetrate the subject. As a result, soft X-rays contribute to patient radiation dose without providing useful information. In order to reduce the radiation dose to a patient, X-ray filters that block soft X-rays were developed. Due to differences in attenuations between the central and peripheral regions, commercial CT scanners are commonly equipped with a beam-shaping filter or bow-tie filter instead of the flat filter [22, 23]. In addition, specific beam-shaping filters that adapt to different examinations such as head, body, and cardiac were developed [24]. One possibility for further reducing patient dose is to use dynamic bow-tie filters which modify X-ray beam as a function of view angle [25, 26].

#### 2.1.2. Collimator

Since the focal spot in the X-ray tube is not truly a point source, beam coverage in some regions is partially irradiated. These regions are called penumbra. In multislice CT, overbeaming caused by focal spot penumbra does not contribute to image reformation. As a result, exposure to such radiation is unnecessary. In multislice row CT, thick beam collimation has better radiation utilization [27]. This is because radiation dose due to overbeaming constitutes a smaller percentage of total absorbed doses. However, thick collimation only allows reconstruction of thick sections. To reconstruct thin sections, thin collimator that reduces radiation utilization is required. This indicates that beam collimation should be selected based on the requirements of clinical examinations.

In multislice CT, spiral scan is the commonly used scanning mode which results in overscanning or overradiation [28, 29]. To obtain sufficient data for reconstructing image at the beginning and end of the *z*-axis scan range, at least an additional one-half of a gantry rotation is prerequisite. This so-called overscanning contributes to patient dose but does not contribute to image reformation. Therefore, a hardware-based solution that uses the dynamic or adaptive collimator to block the pre- and postspiral radiation was introduced and used in clinical practice [30, 31].

#### 2.1.3. Detector

In a CT system, X-ray detector is probably the most important component that converts incident X-ray beam into digital signals. In the 1980s, xenon-gas ionization chamber detectors were commonly used in CT. However, with improvements in technology, xenon-gas detectors are replaced by solid-state detectors (e.g., gadolinium oxysulfide) which have higher quantum efficiency. In normal-dose CT examinations, noise from the electronic components of the detector system is negligible and has no significant impact on image quality. However, in low-dose CT examinations, electronic noise becomes one of the most significant factors determining image quality. Hence, it is important to figure out how electronic noise in low-dose scans can be reduced. In recent years, two new CT detectors, GE's Gemstone Detector and Siemens' Stellar Detector, were introduced into the market. Gemstone Detector made from a garnet-based scintillator enables improvements in spatial resolution and signal-to-noise ratio (SNR). Besides, Stellar Detector that combines the photodiode and the analog-to-digital converters (ADC) in one application specific integrated circuit (ASIC) allows a ~20% reduction in electronic noise. As a result, CT scanners equipped with new detectors are expected to have a reduction in patient dose.

Generally, X-ray detectors currently used in clinical CT scanners are energy integrating. In contrast to conventional energy-integrating detectors, photon-counting detectors not only count each photon individually but also record the energy level of each detected photon. Based on the energy-resolving information, photon-counting detectors have the ability to eliminate electronic noise as well as scattered radiation [32, 33]. In addition, photon-counting detectors with energy discrimination capabilities can be used to improve SNR by assigning an energy-dependent weighting factor to each individual photon [34–36]. Due to the improvement of SNR, the patient dose can be further reduced while maintaining acceptable image quality.

In practice, photon-counting detectors are not widely used in CT systems. This is primarily because the fluxes of incident photons are too high. Under high flux irradiation, the pulse resolving time is relatively too long to allow the detector recording each individual photon. As a result, overlapping pulses which lead to a spectral distortion and a loss of counts may occur. This phenomenon is called pulse pileup [37]. Although a clinical CT scanner equipped with photon-counting detectors is not available yet, recent advances in detector electronics and semiconductor detector materials have been contributed to developing a photon counting-based micro-CT system [38–40]. We expect that a photon counting-based CT will soon be ready for clinical study.

#### 2.1.4. Tube Current

Among many scanning parameters, tube current is one of the important factors determining the radiation dose delivered to the patient. Hence, one commonly used technique to reduce radiation exposure is to adapt the tube current according to the patient's size, shape, and attenuation. Methods that automatically modulate the tube current to adapt differences in patient attenuation are known generically as automatic exposure control (AEC) [41–44]. Now, in most modern CT scanners, AEC can perform automatic adjustment of tube current both during tube rotation (i.e., angular modulation) and along the *z*-axis of the patient (i.e., *z*-axis modulation). In angular modulation, the tube current is adjusted to adapt differences in attenuation between anteroposterior and lateral positions. Unlike angular modulation, the *z*-axis modulation adjusts the tube current to accommodate differences in attenuation along the *z*-axis of the patient (e.g., shoulder and abdomen). The main aim of AEC is not only to modulate tube current but also to deliver the right dose in order to achieve adequate image quality for each individual patient and each diagnostic task. The underlying principle of AEC is similar; however, due to different definitions of image quality, AEC systems from four major CT manufactures are implemented somewhat differently [45]. Despite these different implementations, the AEC techniques used in modern CT scanners contribute to a significant reduction in radiation dose to the patient while maintaining the adequate image quality [45].

#### 2.1.5. Tube Potential

To date, lowering tube potential has been demonstrated to improve image quality or reduce radiation dose in several clinical CT exams [46–52]. The underlying principle is based on the fact that iodine has an increased attenuation (i.e., higher contrast) as photon energy decreases toward the *k*-edge energy of iodine. This indicates that for the same radiation dose, the lower tube potential can yield better image quality than the higher tube potential. Alternatively, the improvement in image quality can contribute to reducing patient dose. However, with the increase of the patient size, low kilovoltage (kV) settings would lead to an increase in image noise. Hence, dose reduction using lower tube potential is feasible only if the patient size is below a particular threshold. However, in routine CT scans, it is difficult to determine the optimal kV setting for individual patient, simply because of the complex relationship among kV, mAs, dose, contrast, and image noise. To solve this problem, Siemens recently introduced a novel tool, CARE kV, which allows the optimal kV to be determined automatically for each individual patient and each specific exam. Basically, CARE kV uses information obtained from the topogram to determine the optimal kV which is used to maintain image quality (e.g., contrast-to-noise ratio). Together with AEC described above, this tool can be beneficial in optimizing and reducing radiation dose to each individual patient.

#### 2.1.6. Image Reconstruction

**Figure 2:**
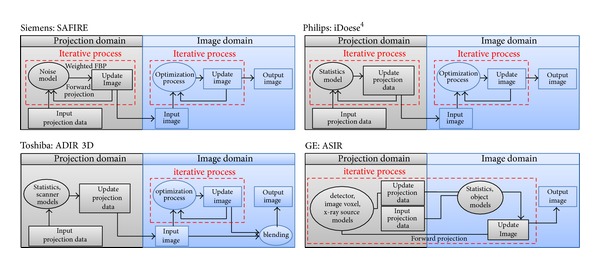
The schematic of four CT manufactures' IR algorithms: Siemens' Sinogram Affirmed Iterative Reconstruction (SAFIRE), Philips' iDose^4^, Toshiba's Adaptive Iterative Dose Reduction 3D (ADIR 3D), and GE's Adaptive Statistical Iterative Reconstruction (ASIR). Both SAFIRE and iDose^4^ have a similar iterative correction process operating in the image domain. In the projection domain, SAFIRE performs a projection data correction via forward and backward (i.e., weighted FBP) projection; however, iDose^4^ directly corrects projection data. ADIR 3D performs a noniterative correction in the projection domain and an iterative correction in the image domain. Also, a weighted blending is applied to the input image (i.e., after projection data correction) and the output image (i.e., after iterative process). Unlike the other three IR algorithms, ASIR performs an iterative correction process by comparing synthesized projection data to measured projection data.

Filtered back projection (FBP) is still the most common reconstruction method used in CT. Over the past two decades, iterative reconstruction (IR) method has been shown to be superior to conventional FBP method in terms of image noise and spatial resolution [53–56]. The improvement in image quality can directly lead to a significant reduction in patient dose. However, IR method is rarely used in routine CT exams, mainly owing to the high computational time required for modeling the system geometry and incorporating physical effects (e.g., noise, scatter, beam hardening, and detector response). Due to advances in hardware [57–59] and renewed emphasis on CT dose reduction, fast IR methods have recently been developed and implemented for radiation dose reduction. Now, IR methods are available on many CT scanners from four major CT manufactures with different algorithms (Figure 2), to perform corrections in both the projection and the image domain. They are applied to achieve noise reduction and artifact reduction as well as to preserve structural edges.

Another important image reconstruction technique toward the goal of reducing radiation dose is the use of incomplete projection data, either to acquire a small number of projection angles or to use the truncated projection data. The first strategy is the sparse-view CT and the second one is the interior CT [60]. With the recent introduction of compressed sensing (CS) in CT [61, 62], it is possible to perform image reconstruction from a small number of projections while still maintaining image quality [63–67]. The basic concept of CS is to recover a signal or an image from measured data with sampling rates below the Nyquist criterion. To accurately restore the signal from the undersampled data, computational load may be still too high for practical use. Thanks to the hardware-accelerated image reconstruction technique [57–59], it is expected that fast CS-based reconstruction methods will be introduced to allow an ultra-low dose CT scan in clinical routine. On the other hand, interior CT uses the projection data passing through a region of interest (ROI) to reconstruct image. In practical, this technique can lead to a dose reduction in patients with cardiac or breast CT scans. The exact ROI reconstruction methods from such truncated projection data have been proposed [68, 69]. Unlike approximate reconstruction methods [70, 71], the new analytical reconstruction method called differentiated backprojection (DBP) was shown to have a potential to exactly solve the ROI reconstruction [60]. Despite the fact that these reconstruction methods have a great potential to reduce CT radiation dose, there are some assumptions needed to be satisfied. For example, some prior knowledge about the object is required.

### 2.2. Dual-Energy CT (DECT)

Due to the recent advances in CT technology including sandwiched detectors [72, 73], rapid kV switching [74], and dual-source and detector [75–77], there is a renewed interest in DECT. To date, DECT has many clinical applications such as bone removal with plaque highlighting, quantification of iodine concentration, visualization of tendons and ligaments, and characterization of renal calculi [78–80]. Using the material-specific information provided by DECT data, virtual noncontrast (VNC) images can be generated by removing iodine content from the dual-energy contrast-enhanced CT images. As a result, DECT has the advantage of omitting one unenhanced (i.e., precontrast) CT scan, thus reducing the overall radiation dose. For example, VNC images generated from either nephrographic- or excretory-phase DECT data may have the potential to replace unenhanced CT images for detection of urinary stone [81, 82], evaluation of haematuria [83] and characterization of renal masses [84, 85]. One recent study showed that iodine map generated from DECT can be used to differentiate between benign and malignant mediastinal tumors [86]. Moreover, iodine concentration provided by DECT strongly correlated with the maximum standardized uptake value of ^18^FDG PET that was commonly used functional imaging technology [87]. More importantly, initial results showed that DECT can be used as an imaging biomarker for predicting the therapeutic benefit in patients with gastrointestinal stromal tumors [88]. We believe that DECT is capable not only of providing functional imaging information but also of giving dose-saving opportunities.

### 2.3. Perfusion CT (PCT)

PCT is an existing imaging technique that has been used to determine tissue perfusion from dynamic contrast-enhanced CT data. Due to the feasibility of rapid acquisition of data (i.e., multislice spiral CT) and the availability of commercial software, clinical applications of PCT in acute stroke, cardiology, and oncology have continued to increase [89, 90]. Recent results showed that PCT positively correlated with tumors in the study of animal models [91, 92]. With PCT, it is also possible to detect and predict tumor response to chemotherapy and radiation therapy [93]. To obtain perfusion parameters (e.g., blood flow and volume and vascular permeability), sequential acquisition of CT data after a bolus injection of contrast agent is required. Typical image acquisition time is about 30 seconds or longer, depending on organ or tissue being scanned, so the radiation dose of a PCT scan is much higher than that of a routine CT scan [94]. As a result, methods that allow for a significant reduction of CT dose without degrading image quality are highly desirable. Since PCT contains temporal information, reconstruction algorithms that incorporate temporal filtering techniques have been proposed to reduce image noise [95–99]. Indeed, such reconstruction algorithms can allow a significant reduction in image noise, thereby having a large potential to reduce the radiation dose in PCT. One alternative strategy to reduce radiation dose in PCT is to optimize scanning parameters such as total scanning time and sampling interval [100, 101]. With the implementation of dose-reduction technologies, PCT will be increasingly used in clinical practice.

### 2.4. Summary

We would like to summarize the CT dose reduction technologies so that it would be easier to understand their feasibilities.

- To accomplish the principle of ALARA, each CT scan should be customized for the patient's age, weight, size,and imaging task. Individual customized CT scanning techniques used in current clinical CT scanners include examination-specific bow-tie filters, adaptive collimator, and automatic tube potential selection with tube current modulation.
- CT detectors with integrated electronics or a garnet-based scintillator have a great potential to reduce noise, thereby improving image quality and dose efficiency. CT scanners equipped with high-rate photon-counting detectors will be available in the near future for further dose reduction.
- As provided by four major CT manufactures, iterative reconstructions result in significant dose reduction and improved image quality over conventional filtered back projection reconstruction methods. New reconstruction methods for sparse-view CT and interior CT will be an alternative to dose reduction.

## 3. Imaging Acceleration in Magnetic Resonance Imaging

Since the introduction of MRI in the early 1970's [102], its techniques have been much improved from hardware to image reconstruction. Due to the intrinsic physical limitation of MRI, the speed of data acquisition is always the problem comparing to CT. Hence, a number of acceleration techniques have been developed over the past four decades and the shortened acquisition time has greatly expanded clinical applications of MRI, especially for dynamic or time-resolved MRI, such as perfusion imaging [103–106], contrast-enhanced MR angiography [107–111], functional MRI [112, 113], and cardiac function examinations [114–117]. Those acceleration techniques could be divided into two categories: parallel imaging and dynamic acceleration. Besides, CS, which is beyond the limitation of Nyquist criterion [61, 62], inspires the MR physicists and the radiologists for even faster scans.

### 3.1. Parallel Imaging

**Figure 3:**
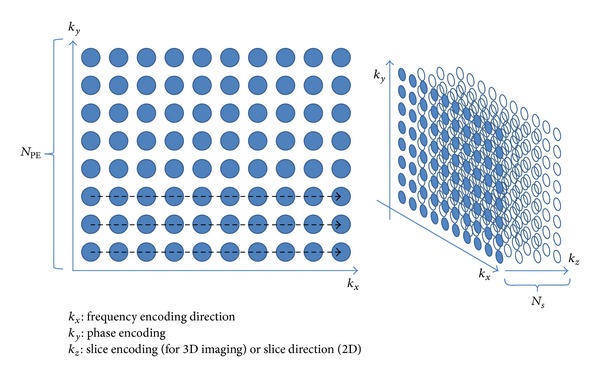
Illustration of three-dimensional *k*-space in MRI. Generally, the sampling time of *k*_*x*_ was less than repetition time (TR), so the acquisition time of a set of image could be written as TR × *N*_PE_ × *N*_*S*_.

The typical acquisition time of an MR image is the product of the repetition time (TR), number of phase-encoding lines (*N*_PE_), and the slice number (*N*_*S*_) (Figure 3). In order to shorten the scan time, the reduced phase-encoding *k*-space lines are generally acquired, which would either sacrifice the image resolution or result in the aliasing artifacts. Parallel imaging technique, which combines the design of the phase-arrayed coil and various image reconstruction algorithms, makes the faster data acquisition possible. Hence, the common features of the parallel imaging include the under-sampled *k*-space data and the phase-arrayed coils, which provided additional spatial information for image reconstruction to solve the aliasing patterns. The reconstruction algorithms could be divided into SENSE and GRAPPA family, where the undersampled data are reconstructed in image domain and *k*-space, respectively. Besides, the multislice acceleration acquisition is a kind of extension of parallel imaging and we would discuss it after the two conventional techniques.

#### 3.1.1. Generalized Autocalibrating Partially Parallel Acquisition (GRAPPA)

GRAPPA [118] technique originated from SMASH [119]. SMASH technique reconstructed the MR images from filling in the missing phase-encoding lines by using the phased-array coil, whose elements are parallel to the phase-encoding direction. Because the acquired signals from different surface coils imply the spatial sensitivity information in the meantime, they could be used to generate multiple datasets, which are called harmonics, by using linear combination with different weightings to complete the whole *k*-space. The restored full *k*-space was then Fourier-transformed to obtain the reconstructed image (Figures 2 and 3 in [119]). Depending on the concept of linear algebra, *N* components could form maximal *N* linearly independent combinations and result in maximal acceleration rate of *N*. However, the harmonics are generally less than the total number of array components due to the geometrical constraints.

**Figure 4:**
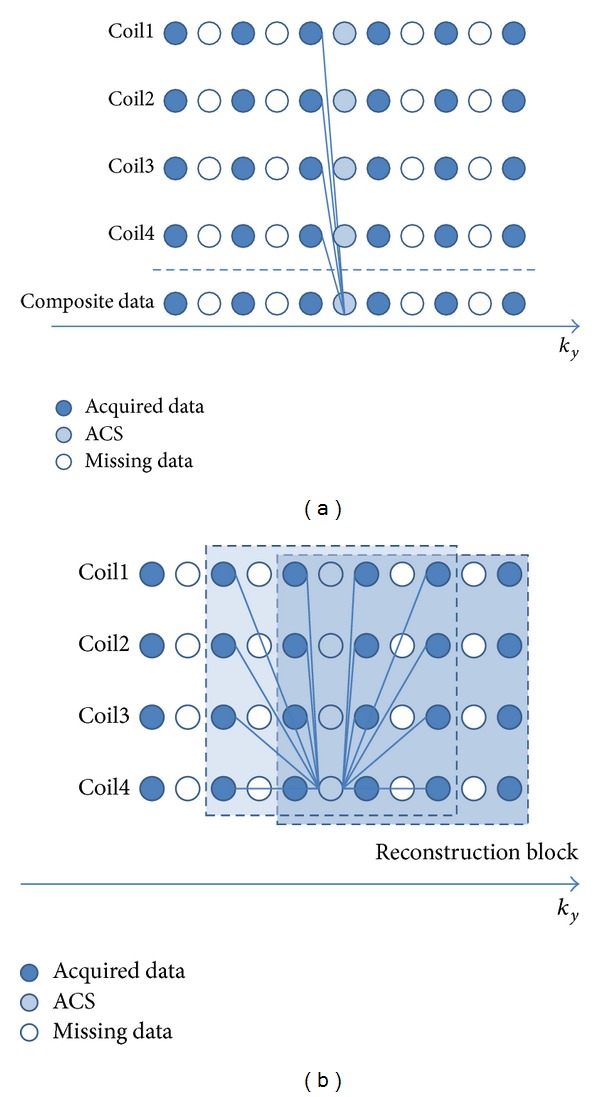
(a) AUTO-SMASH or VD-AUTO-SMASH reconstruction process for the acceleration rate of 2. Each *k*-line in a single coil was composited to fit the ACS line, and the relationship was applied to fill in the missing *k*-lines. The procedures constructed the whole *k*-space and then the reconstructed images were obtained. SMASH did not additionally acquire ACS. It used the estimated coil sensitivities to composite the missing data. (b) GRAPPA used multiple lines from all coils to fit a line in one coil. The reconstructed images from each coil were obtained after the *k*-space acquired from each coil was fully restored. The uncombined images were merged using sum of squares or other adaptive algorithms. The concept of reconstruction block made GRAPPA flexible using different “blocks” to fit the ACS lines for better performance. For example, at least there were three blocks used for ACS lines fitting, which would provide a more accurate outcome.

However, the major pitfall of SMASH is its high dependence on accurate coil sensitivity function, which might be easily affected by coil positions, subjects, and magnetic field inhomogeneities. Hence, some other methods accompanying the acquisition of autocalibration signals (ACS) near the central *k*-line were developed for the sensitivity function calibration, such as AUTO-SMASH [120] and VD-AUTO-SMASH [121]. Furthermore, GRAPPA provided a more general form to reconstruct the full *k*-space data using multiple blocks in sliding manners rather than SMASH and its analogies from adjacent acquired *k*-lines (Figure 4). It is more flexible to fit the ACS from the acquired signals and restores the missing *k*-space data more precisely. The number of ACS is another factor to influence the reconstruction quality. Generally speaking, the more ACS acquired, the more accurate GRAPPA weightings obtained, which result in less artifacts but reduced the acceleration efficiency. GRAPPA is a more robust method and less relies on the coil sensitivity maps, so that the cardiac, lung, and abdominal applications become feasible. Besides, it could be also applied to 3D imaging [122, 123].

#### 3.1.2. Sensitivity Encoding (SENSE)

**Figure 5:**
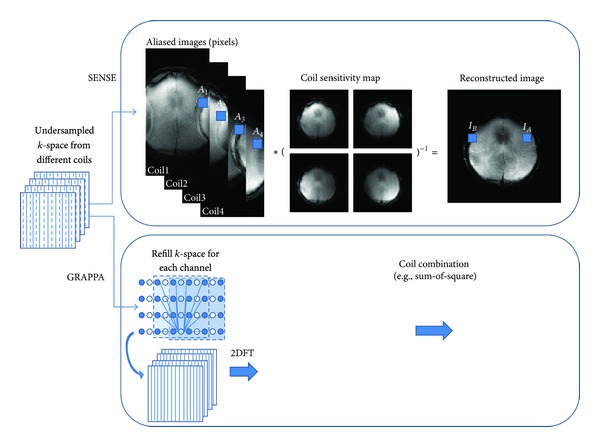
The reconstruction process of SENSE and GRAPPA. The example used a four-channel array coil for the acceleration rate of two. Each of the aliased pixels (*A*_1_, *A*_2_, and so on) came from *I*_*A*_ and *I*_*B*_ with different weightings depending on the coil sensitivity values. It could be represented as *A*_*i*_ = *I*_*A*_ *C*_*Ai*_ + *I*_*B*_ *C*_*Bi*_, where *A* was the aliased pixel, *I* was the intensity of the unaliased pixel, and *C* was the coil sensitivity weighting from the corresponding location. It could be possible to reconstruct the data by finding the inverse matrices of coil sensitivity maps, which could be written as *AC* ^−1^ = *I*. GRAPPA reconstructed the undersampled *k*-space using ACS fittings to calculate the GRAPPA weightings and refill the missing lines from each coil. Each restored full *k*-space was transformed to obtain the single-coil images, which were then combined together to finalize the reconstruction.

SENSE [124] technique began from the aliased images resulting from the under-sampled *k*-space acquisition. Considering the case of acceleration factor *R*, each aliased pixel from different phase-arrayed coils should be divided into *R* partitions with different weightings relating to coil sensitivities (Figure 5). Hence, the coil sensitivity map, which is usually obtained from a low-resolution prescan at the beginning of the image acquisition, is the prior information for SENSE reconstruction. Since SENSE reconstruction much relies on the coil sensitivity map, the inaccurate sensitivity map would lead to residual aliasing artifacts. One of the most important factors to sensitivity map is SNR. Low SNR regions, for example, lung images, have a difficulty in determining the coil sensitivity distribution.

The common features for the two parallel imaging families are the reduction of SNR and the residual aliasing. The significant difference is the reconstruction domain: *k*-space for GRAPPA and image domain for SENSE (Figure 5). The SNR reduction of parallel imaging could be generalized to

(1) $$\begin{array}{c} {\text{SNR}}_{\text{PI}}=\frac{{\text{SNR}}_{\text{fs}}}{g\sqrt{R}}, \end{array}$$

where fs means full sampling, *R* is the acceleration factor, and *g* is the factor relating to some coil properties, such as geometry design [125, 126]. The meanings of *g*-factor in GRAPPA and SENSE are different, but it is mostly larger than one. Hence, many dedicated surface coils were developed, such as breast coils, spine arrays, and head coils, to reduce the noise enhancement. In addition, the ill-conditioned problem also leads to the loss of SNR. Thus, the use of regularization in SENSE [127–129] as well as GRAPPA [130] was developed. Though there are some compromises in parallel imaging, it is still routinely used in various clinical applications [131] due to improvements in acquisition time, spatial or temporal resolution, and image quality such as reduction of motion artifacts and EPI geometric distortion.

#### 3.1.3. Multislice Excitation

**Figure 6:**
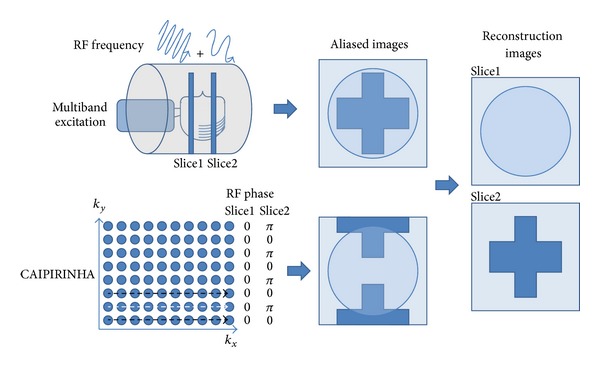
Schematic description of the differences between MB excitation and CAIPIRINHA. The slice selection gradient would be applied during the RF excitation, where the RF frequency and the slice resonant frequency were the same. Since MB excitation and CAIPIRINHA excited multiple slices simultaneously, the bandwidth of RF excitation would become wider than the single slice excitation. MB excitation modulated the RF excitation pulse with several frequencies to excite multiple slices, so the acquired images would overlap together without FOV shifting (the upper row). Considering the bandwidth of frequency encoding and the removal of the artifacts, the excitation slices should have gaps with several centimeters depending on the gradient strength. Besides, the pixel skew was another factors controlled by the gradient strength [20]. CAIPIRINHA, which also excited multiple slices simultaneously, controlled the aliasing pattern by phase-modulated RF pulses. In the figure, for example, the phase of RF pulse in the second slice was interlacedly altered to shift the overlapping patterns with FOV/2 (the lower row). The black dashed arrow indicated the phase-encoding line without phase alteration in both slices, and the white one did the phase alteration of *π* only in the second slice. It could improve the *g*-factor to increase the SNR in image reconstruction. The technology could get rid of the pixel skew in MB excitation and make adjacent slice excitation feasible.

GRAPPA and SENSE could be extended to 3D imaging [122, 123], which is slab excited without additional RF modification. An alternative idea for acceleration is the multislice simultaneous excitation with the sharing data acquisition schemes. The RF excitation type could be divided into frequency modulation, such as multiple band (MB) excitation [20, 132], and phase cycling, such as CAIPIRINHA [133] (Figure 6).

Both techniques have some intrinsic limitations. In MB excitation, the frequency offsets between slices should be at least equal to or larger than the slice bandwidth to ensure the successful reconstruction from the slice aliased images. Thus, two solutions could be applied: either to increase the gap between the excited slices at around several centimeters or to strengthen the slice selection gradient, which would result in the pixel skew. CAIPIRIHNA, on the other hand, controlled the aliasing pattern using phase modulated multislice RF excitation pulses to avoid the pitfalls in MB excitation technique and improved the image quality of 2D-GRAPPA or 2D-SENSE reconstruction resulting from *g*-factor related noise enhancement (Figures 6 and 7 in [133]). Furthermore, instead of providing phase modulated RF pulses, the shifted aliasing patterns could perform more easily in 3D volume images via changing the encoding strategy (2D CAIPIRINHA) [21]. Hence, various developments in sequences [134, 135] and clinical applications [136, 137] were demonstrated more easily. However, neither CAIPIRINHA nor conventional MB excitation techniques are not applicable to single-shot echo planar imaging (EPI), since the former method uses shot-by-shot RF modulation and the latter one suffers from high *g*-factors due to the hardware limitations. Hence, taking the concept of inclined readout [20, 132], the blipped wideband [138] and blipped-CAIPIRINHA [139] were proposed to make simultaneous multislice EPI and diffusion imaging feasible with reasonable *g*-factors. The significant advantages are that the temporal resolution could be increased for fMRI and the acquisition time could be much reduced for DSI or Q-ball imaging.

MB and CAIPIRINHA, generally in charge of the slice direction acceleration, could incorporate with the conventional parallel imaging techniques to fully achieve 2D acceleration in a better performance. Since all the techniques are based on the reduced *k*-space sampling for acceleration, the residual artifacts should be carefully dealt with.

### 3.2. Dynamic Acceleration

The dynamic MRI introduces temporal information into the acquisition, so it provides an additional dimension for acceleration. Only the signals of certain regions were varied with time in dynamic imaging; for example, contrast-enhanced angiography shows the signal intensity change in vessels and the rest parts are almost static. Hence, there are lots of redundant acquired data during the examination, and it could be well manipulated to reduce the scan time for each time frame and to increase the temporal resolution. The first idea came to view sharing in *k*-space.

#### 3.2.1. View Sharing

Taking the advantages of the similarity of the adjacent time frames, several view sharing technologies were developed since the late 1980s, including MR fluoroscopy [140], Keyhole [141], BRISK [142], and TWIST [143, 144]. The major differences among the technologies are the manners on dynamical *k*-space data replacement and the data recovery.

**Figure 7:**
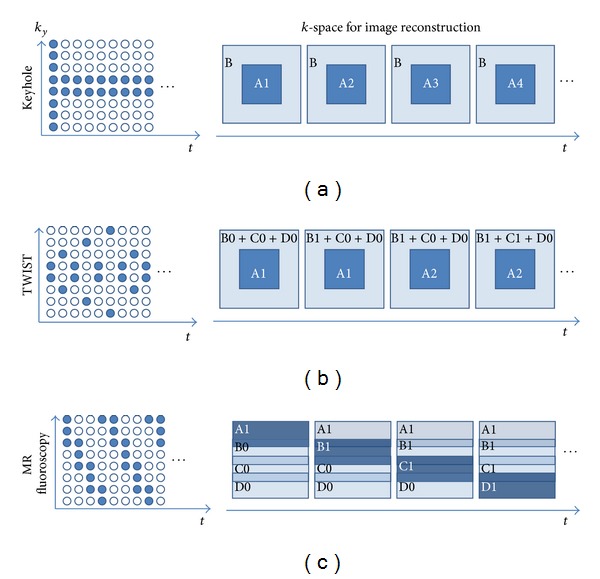
The sampling features in view-sharing technologies: Keyhole, TWIST, and MR fluoroscopy. (a) Keyhole acquired full *k*-space at the beginning or the end of the dynamic scan. Only central *k*-space (A1, A2, and so on, where the number indicated the times of update) was renewed at each time frame and the rest was kept the same for image reconstruction. (b) TWIST updated the central *k*-space information more frequently. The outer *k*-space was divided into several subregions, for example, three (B, C, and D) in this case, and only one of them was updated at each time frame interleaved by the renewal of the central *k*-space. (c) MR fluoroscopy updated the *k*-space sequentially and uniformly everywhere. Part of the information was acquired at each time frame and updated to *k*-space for dynamic image reconstruction.

MR fluoroscopy periodically updates the *k*-space data so that the refreshed rate of each part is the same. Because partial *k*-space is acquired at each time point, the data recovery should include the information from some previous adjacent time frames and the latest acquired one to obtain full *k*-space data and then convert to images. The technique is also called moving average or sliding window reconstruction. However, it is not efficient to update the *k*-space uniformly due to most of the useful information locating on central *k*-space. Hence, Keyhole pushes the idea into a limit, where the peripheral *k*-space data are only acquired once at the beginning or the end of the dynamic scan and the center is refreshed at every time frame. BRISK and TWIST are the compromise between the two extreme cases. They divide *k*-space into different zones, where the central one and the one of the peripherals are alternatively updated (Figure 7).

These techniques refill the missing *k*-space using the temporally closest data. The process is equal to the dynamic images interpolation, which would smooth the dynamic response curves similar to applying low-pass filters. Hence, some high temporal frequency information would get lost, so that the acceleration rate and the view-sharing methods should be carefully chosen depending on different applications. However, they did not really take the advantages from the additional dimension, time, for acceleration, so more sophisticated approaches were developed.

#### 3.2.2. Unaliasing by Fourier-Encoding the Overlaps Using the Temporal Dimension (UNFOLD)

**Figure 8:**
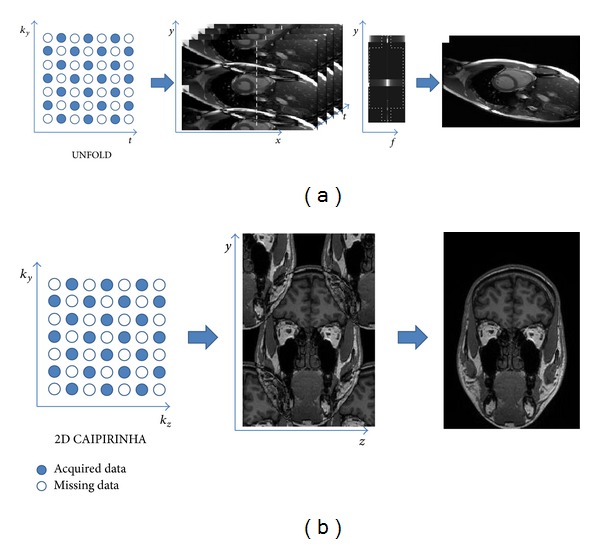
The similarity and difference between UNFOLD and 2D CAIPIRINHA. Both techniques used two-dimensional accelerations, where the second domain in UNFOLD is time and that in 2D CAIPIRINHA is the slice encoding direction (*k*_*z*_). (a) UNFOLD transformed the dynamic aliased images to the *x*-*f* space, where the information was more concentrate. The *x*-*f* aliasing patterns could be shifted by changing the sampling patterns. Hence, the space was suitable to reduce the aliasing patterns and reconstruct the images by applying appropriate filters (the dashed cross region). (b) 2D CAIPIRINHA also changed the sampling patterns to shift the aliased images, which could reduce the *g*-factor and improve the SNR in reconstruction. The performance would be better than that in conventional 2D parallel imaging techniques [21].

UNFOLD [113] was the very early technique taking the advantages of spatial and temporal information for acceleration. It converted the acquired *k*-space data with temporal information (*k*-*t* space) into *x*-*f* space to observe the dynamic patterns, so that we could more effectively exploit the *x*-*f* space for higher acceleration rates (Figure 8(a)). The “*x*” in *x*-*f* space represents the image domain, not exactly the *x*-axis, and generally the undersampled *k*-space is along the phase-encoding direction, which is usually the *y*-axis. The concept of UNFOLD is similar to 2D CAIPIRINHA [21], where the images become less aliasing by modifying the sampling strategy (Figure 8(b) and Figure  3 in [21]). The major difference is the second acceleration dimension of 2D CAIPIRIHNA along slice encoding direction and that of UNFOLD temporally. However, a prerequisite of UNFOLD technique is that the aliased *x*-*f* space should not overlap in major frequency components. Then the aliasing artifacts could be removed in *x*-*f* space by applying appropriate filters to fully reconstruct the images.

The limitation of acceleration rate in UNFOLD depends on different applications. The larger the portion of dynamic region within an FOV, the less the acceleration rate achieve. Hence, the acquisition time of fMRI could be more efficiently reduced than that of cardiac imaging.

#### 3.2.3. *k*-*t* Family

**Figure 9:**
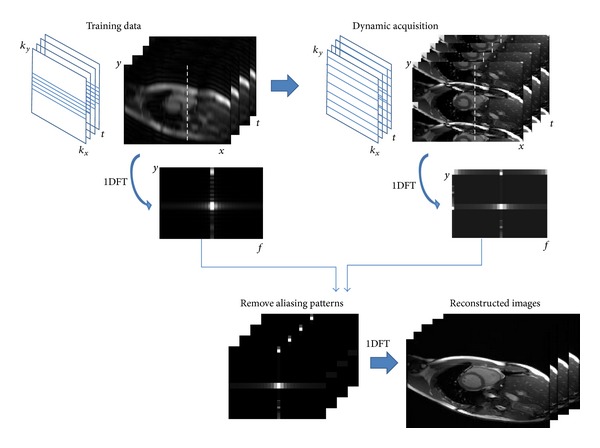
*k*-*t* BLAST for dynamic imaging acceleration. The data acquisition is divided into two parts, training data (low resolution images) and dynamic acquisition (undersampled *k*-space images). The *x*-*f* space of the training data is used to preliminarily understand the features of the dynamic scan, and then the undersampled data could be involved for image reconstruction without applying any determinate filters. An alternative method was to embed the central *k*-line acquisition in every time frame and the data were split into two parts as the description above when doing image reconstruction.

Depending on the concept from UNFOLD technique, *k*-*t* BLAST and *k*-*t* SENSE [145] were developed to enhance the robustness of the achievable acceleration by acquiring the central part of *k*-space at the beginning as the training data before the undersampled dynamic *k*-space acquisition. Hence, the whole acquisition is divided into two parts: the training scan, providing the low spatial resolution images without aliasing artifacts, and the undersampled scan, containing the full spatial and temporal information but with fold-over artifacts. *k*-*t* BLAST and *k*-*t* SENSE use the training data as prior information to understand how to resolve the aliasing patterns and to obtain the fully-reconstructed dynamic images. *k*-*t* SENSE, combining the parallel imaging technology, was an extension of *k*-*t* BLAST, and both technologies reconstruct the images in *x*-*f* space (Figure 9). Because *k*-*t* methods introduce temporal information to increase the degree of freedom, their performance, especially in noise reduction of the reconstructed images could be much better than the traditional parallel imaging at high acceleration factors.

*k*-*t* GRAPPA [146, 147] reconstruct the data directly in *k*-*t* space without transforming to *x*-*f* space. ACS is acquired to determine the weightings for *k*-space reconstruction as GRAPPA does. *k*-*t* ^2^ GRAPPA [148] is an extension of the series and scatters the central *k*-space acquisition into different time frames. Hence, the reconstruction should firstly use *k*-*t* GRAPPA to refill the undersampled central *k*-space and then to restore the full *k*-space data. *k*-*t* PCA [103] is another kind of extension, which reconstructed the images using principal components (PC), instead of temporal frequency. Thus, it uses *x*-PC space for data reconstruction. Taking the advantages of mathematical properties of principal component analysis (PCA), *k*-*t* PCA could even reduce the artifacts for higher acceleration rates.

### 3.3. Compressed Sensing (CS)

CS [62] was first proposed in the field of information theory and is applied to MRI with the sampling rate beyond the Nyquist criterion. The sampling number could be much reduced in CS, as a result, the acquisition time becomes much shorter. There are three basic requirements for the CS approach, including the sparsity in a known transform domain, the incoherence of the undersampled artifacts and the nonlinear reconstruction.

Most of the MR images are not sparse from image point of view except angiography. However, sparsity is the most fundamental requirement to represent whether the data are compressible or not. Many different kinds of images are sparse in discrete cosine transform (DCT) and wavelet transform, which are the cores of image or video compression technologies. Medical images share the same property of sparsity, so that the DCT and the wavelet transform are both good for brain and angiogram which were capable to use only 5% to 10% largest transform coefficients for reconstruction [149]. Conventional undersampled *k*-space was coherent, that is, the sampled *k*-space was skipped periodically and the reconstructed images resulted in regular aliasing patterns. On the other hand, the incoherent undersampled *k*-space would lead to the noise-like artifacts, so the dealiasing problems could be converted to a denoise problem. The two aliasing patterns show significant differences in Figure 1 of [150]. Since the sampling strategy has destroyed the linear transformation relations between two domains (*k*-space and image), some nonlinear reconstruction methods should be applied to iteratively approach the solutions, such as conjugate gradient method [151].

The undersamples along frequency encoding direction could not actually reduce the scan time in MRI and it was not feasible to achieve the fully randomized sampled *k*-space. Several encoding schemes, such as radial and spiral [110, 152, 153], could approach the variable-density sampling, which results in more incoherent aliasing patterns. For morphological or static images, 2D imaging acquisition is not preferred because the randomly undersampled dimension is limited. It is more attractive on three-dimensional imaging or dynamic images, because there are two nonfrequency encoding planes (*k*_*y*_-*k*_*z*_ or *k*_*y*_-*t*), which would achieve random undersampling more easily. However, one of the features of *k*-space should be taken into consideration when doing randomized undersampling: the energy of *k*-space is concentrated in the central region. Hence, the central *k*-space should be less undersampled than peripheral regions to keep the image quality and to accelerate the data acquisition more efficiently.

CS has been demonstrated in various applications with the combination of parallel imaging techniques in static images [149, 154, 155]. Furthermore, there were also technologies developed with the combination of the concepts from *k*-*t* BLAST and incoherent sampling, such as *k*-*t* SPARSE-SENSE [156, 157] and *k*-*t* FOCUSS [158], and dynamic MRI using CS was applied to cardiac imaging [159, 160] and fMRI [161, 162]. However, the computational time is still too long for daily clinical use. Thus, the improvement in algorithms and computer hardware, which shorten the postprocessing time, would bring CS more feasible.

### 3.4. Summary

We would like to summarize the MR acceleration technologies so that it would be easier to distinguish the differences and understand their feasibilities.

- Parallel imaging uses the multiple-channel surface-arrayed coils to reduce the *k*-space sampling. The reconstruction methods are divided into GRAPPA and SENSE two families, which are commonly used in clinical routines.
- Multislice excitation, the extension of parallel imaging, could be divided into frequency modulation (MB) and phase modulation (CAIPIRINHA) for the acceleration along the slice direction with better performance.
- Temporal domain is involved in dynamic acceleration methods, such as UNFOLD and *k*-*t* BLAST. UNFOLD applies an appropriate filter to remove the aliasing patterns, where *k*-*t* BLAST does it via the low-resolution training data at the beginning of the dynamic acquisition.
- Compressed sensing MRI brings the acquisition beyond the Nyquist criterion limitation both in static and dynamic images.
- All the technologies above are compatible to parallel imaging techniques.

With the appropriate combination of these techniques, we could foresee the huge leap in MR acceleration in the near future.

## 4. Conclusion

CT dose consideration and MR acquisition could be viewed as the two obstacles during their development. We have seen many techniques developed to solve the problems, to broaden their applications, and to push the diagnostic clinical imaging into molecular imaging level. Besides, their intrinsic superiorities are kept to measure the physiological parameters, such as blood flow, blood volume, and *T*_1_/*T*_2_ parameters or diffusion imaging specified by MRI. The information has been investigated for oncology imaging, cancer treatment followup, neurodegenerative diseases, and so on. Due to the growing knowledge of the human multiple “-omes,” such as genome, proteome, and metabolome, more information is included to analyze the correlation between the -omic data and the clinical imaging. It potentially does great benefits to the early detection, staging, and treatment response of the diseases. As the personal information is involved in the clinical procedures, it comes closer to realize the personalized medicine. The development strongly depends on the conjunction between various fields, including diagnostic imaging, nanotechnologies, and -omics database analysis.
